# Evaluating the psychosocial burden and unmet needs for health care access among older adults with inflammatory skin disease

**DOI:** 10.1016/j.jdin.2024.11.006

**Published:** 2024-11-30

**Authors:** Alyssa M. Roberts, Danielle West, Benjamin Stroebel, Katrina Abuabara

**Affiliations:** a Department of Dermatology, University of California San Francisco, San Francisco, California; b Department of Epidemiology & Biostatistics, University of California, San Francisco, California; c John A. Burns School of Medicine, University of Hawaii at Manoa, Honolulu, Hawaii; d Department of Epidemiology and Biostatistics, School of Public Health, University of California Berkeley, Berkeley, California; e Department of Physiological Nursing, University of California San Francisco, San Francisco, California

**Keywords:** access, alopecia areata, anxiety, atopic dermatitis, burden, health and retirement study, inflammatory skin disease, isolation, loneliness, psoriasis, psychology, vitiligo

*To the Editor:* Inflammatory skin disease (ISD) is prevalent among older adults,^1^ but there are minimal data on the psychosocial burden of ISD and health care access among this population. Focused study on these areas in older adults is crucial given the growing aging population and their unique characteristics.

We performed a cross-sectional analysis of data from the Health and Retirement Study (HRS), an ongoing, nationally representative survey of US adults over age 50, to explore the psychosocial burden of ISD and identify unmet needs for health care access among older adults. In 2019, the HRS included questions specifically about ISD, and the supplementary questionnaire had an 83% response rate. Our final sample included 4774 respondents that completed all study components.

ISD was defined as a self-reported physician diagnosis of psoriasis, atopic dermatitis, alopecia areata, and/or vitiligo. These conditions were selected based on the data available through the HRS. Psychosocial burden was recorded through individual measures of life satisfaction, subjective well-being, participation in activities, and isolation. Component scores were aligned so that higher values indicated less psychosocial burden. These scores were converted to standardized z-scores (mean = 0; SD = 1), which were averaged to obtain a composite score. Linear regression models were used to examine relationships between psychosocial scores, respondent characteristics, and ISD. Barriers to care were identified through self-reported trouble finding a specialist. Logistic regression models were used to examine the association between trouble finding a dermatologist and ISD. To allow for comparison with other systemic inflammatory conditions in the HRS, respondents with inflammatory bowel disease (IBD) and rheumatoid arthritis (RA) were also analyzed.

ISDs were reported in 12.6% of respondents and were more common among females than males (OR = 1.58, 95% CI = 1.26-1.99) and less common in Black than White respondents (OR = 0.67, 95% CI = 0.50-0.89). ISDs as a group were significantly associated with worsened isolation prior to adjustment via the Holm-Bonferroni method (beta = −0.101, *P* = .046, adjusted *P* = .230). None of the individual ISDs were significantly associated with composite psychosocial outcome (Table I). IBD (beta = 0.117, *P* = .508) and RA (beta = −0.089, *P* = .071) also lacked significant disease associations with composite psychosocial score. ISD respondents were more likely to report trouble finding a dermatologist (adjusted OR [aOR] = 2.54, 95% CI = 1.15-5.26), although the absolute proportion was low (1.83%). For comparison, IBD and RA respondents did not report significant trouble finding a gastroenterologist (aOR = 1.25, 95% CI = 0.20-4.39) or rheumatologist (aOR = 1.41, 95% CI = 0.58-3.15), respectively. Of the ISD respondents who endorsed difficulty when seeking a specialist, 34.4% reported delaying care due to cost (Fig 1). When asked what non-cost-related difficulty was encountered when seeking a specialist, 30.6% cited a lack of appointments.

**Table I:** Multivariate analyses of inflammatory skin disease and composite and component psychosocial outcomes

	Freq (%)	Composite psychosocial score∗^,^†			Life satisfaction∗^,^‡			Subjective well-being∗^,^§			Participation in activities‖^,^¶			Isolation∗^,^#		
		Beta	*P* value	*P* value (adj)	Beta	*P* value	*P* value (adj)	Beta	*P* value	*P* value (adj)	Beta	*P* value	*P* value (adj)	Beta	*P* value	*P* value (adj)
Any ISD	600 (12.6)	−0.043	.380	1	−0.039	.422	1	0.004	.993	1	0.037	.472	1	**−0.101**	**.046**	.230
Psoriasis	177 (3.71)	−0.047	.326	1	−0.050	.295	1	0.028	.574	1	−0.026	.611	1	−0.044	.370	1
Atopic dermatitis	367 (7.69)	−0.030	.532	1	−0.041	.390	1	0.001	.975	1	0.031	.536	1	−0.079	.109	.436
Alopecia areata	46 (0.96)	0.027	.583	1	−0.016	.736	1	0.081	.108	.540	0.024	.638	1	−0.043	.389	1
Vitiligo	113 (2.37)	0.327	.881	1	0.001	.868	1	−0.017	.733	1	0.016	.759	1	0.022	.667	1

The adjusted *P* values were calculated using the Holm-Bonferroni method and are listed as 1 when the penalty is large. Bolded values denote statistical significance (*P* < .05). All models were adjusted for age, sex, race/ethnicity, educational degree, household net worth, body mass index (BMI), smoking status, and alcohol use.

*adj*, Adjusted *P* values; *Freq*, frequency; *ISD*, inflammatory skin disease.

∗ Higher scores indicate improved psychosocial status.

† Composite psychosocial score was obtained from the individual measures (life satisfaction, subjective well-being, participation in activities, and isolation). Results for participation in activities with the Victoria Longitudinal Study activity questionnaire were inverted so that higher scores indicated improved psychosocial well-being (in alignment with the other psychosocial measures). The composite psychosocial score was created by averaging standardized z-scores (mean = 0; SD = 1) from each individual measure.

‡ Life satisfaction was measured with the five-item Diener Satisfaction with Life Scale.^2^ Scores ranged from 5 to 35.

§ Subjective well-being was measured with the six-item Ryff Scale of Psychological Well-Being.^3^ Scores ranged from 6 to 36.

‖ Lower scores indicate improved psychosocial status.

¶ Participation in activities was measured with the extended Victoria Longitudinal Study activity questionnaire.^4^ Scores ranged from 21 to 147.

# Isolation was measured with the three-item scale developed by Hughes et al^5^ for use in large-scale surveys. Scores ranged from 3 to 9.

**Fig 1:**
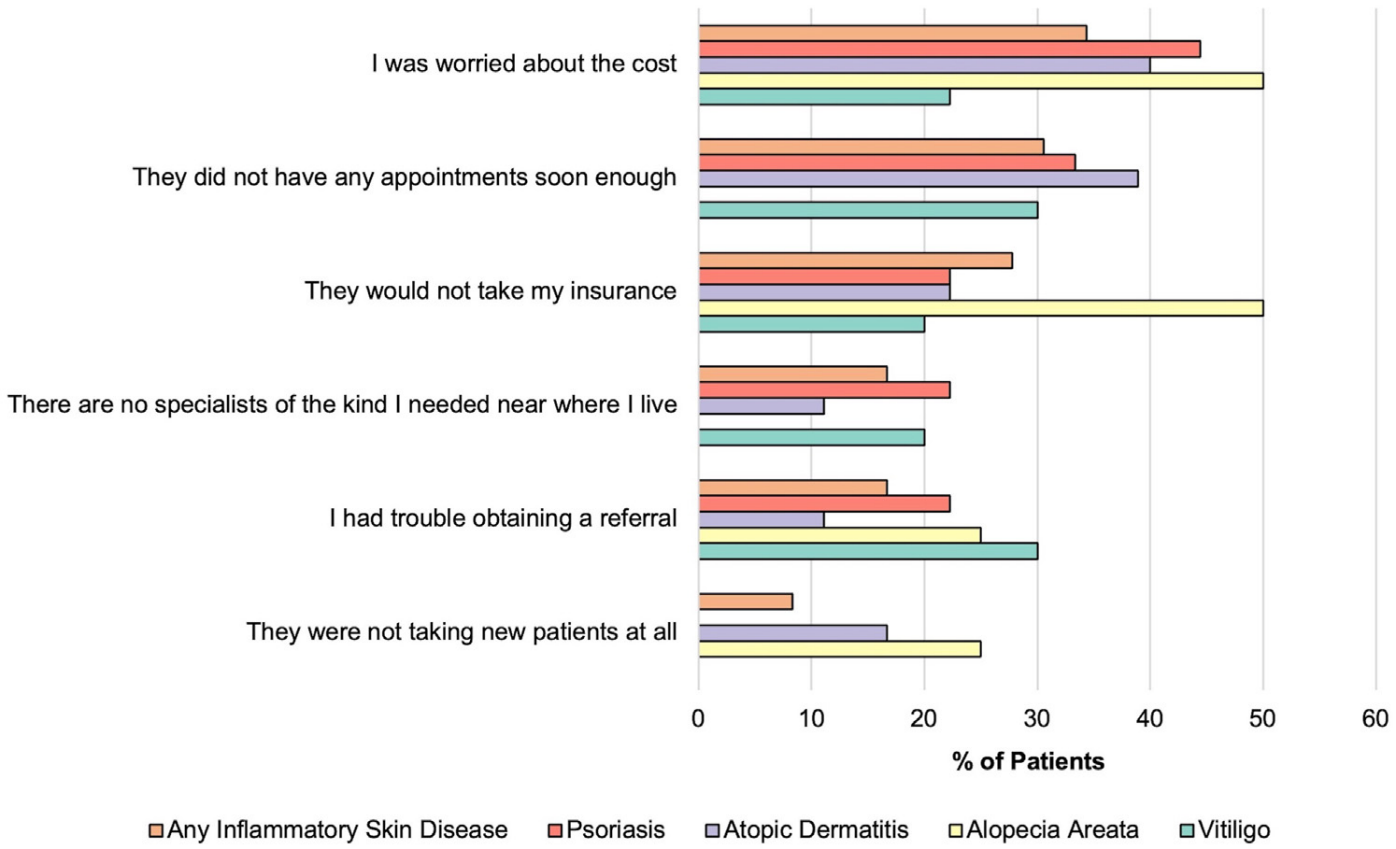
Difficulties reported by inflammatory skin disease patients when seeking a specialist in the last 12 months.

In summary, ISD was prevalent among a nationally representative sample of older adults and potentially associated with increased isolation but not other psychosocial outcomes. These results may underestimate disease burden as we were unable to stratify by disease severity or activity. Moreover, participants reported financial and logistical challenges to accessing dermatologic care. Health care providers should recognize that ISDs are common among older adults and may lead to isolation. Further exploration into cost-effective, accessible care options may help to reduce barriers to care.

## Conflicts of interest

Dr Abuabara reports consulting/advising fees from Amgen, Astria, Incyte Derm, Nektar Therapeutics, TARGET RWE, and Sanofi; and grants to her institution from Pfizer and Cosmetique Internacional SNC.
